# Development of a Physiologically Based Computational Kidney Model to Describe the Renal Excretion of Hydrophilic Agents in Rats

**DOI:** 10.3389/fphys.2012.00494

**Published:** 2013-01-24

**Authors:** Christoph Niederalt, Thomas Wendl, Lars Kuepfer, Karina Claassen, Roland Loosen, Stefan Willmann, Joerg Lippert, Marcus Schultze-Mosgau, Julia Winkler, Rolf Burghaus, Matthias Bräutigam, Hubertus Pietsch, Philipp Lengsfeld

**Affiliations:** ^1^Computational Systems Biology, Bayer Technology Services GmbHLeverkusen, Germany; ^2^School of Engineering and Science, Jacobs University gGmbHBremen, Germany; ^3^Clinical Pharmacokinetics, Bayer Pharma AGBerlin, Germany; ^4^Quantitative Pharmacology, Bayer Pharma AGElberfeld, Germany; ^5^Institut für Radiologie, CharitéBerlin, Germany; ^6^Magnetic Resonance and Computed Tomography Contrast Media Research, Bayer Pharma AGBerlin, Germany; ^7^Global Medical Affairs, Radiology and Interventional, Bayer Pharma AGBerlin, Germany

**Keywords:** kidney, contrast media, osmotic diuresis, viscosity, modeling, simulation, physiologically based pharmacokinetic modeling

## Abstract

A physiologically based kidney model was developed to analyze the renal excretion and kidney exposure of hydrophilic agents, in particular contrast media, in rats. In order to study the influence of osmolality and viscosity changes, the model mechanistically represents urine concentration by water reabsorption in different segments of kidney tubules and viscosity dependent tubular fluid flow. The model was established using experimental data on the physiological steady state without administration of any contrast media or drugs. These data included the sodium and urea concentration gradient along the cortico-medullary axis, water reabsorption, urine flow, and sodium as well as urea urine concentrations for a normal hydration state. The model was evaluated by predicting the effects of mannitol and contrast media administration and comparing to experimental data on cortico-medullary concentration gradients, urine flow, urine viscosity, hydrostatic tubular pressures and single nephron glomerular filtration rate. Finally the model was used to analyze and compare typical examples of ionic and non-ionic monomeric as well as non-ionic dimeric contrast media with respect to their osmolality and viscosity. With the computational kidney model, urine flow depended mainly on osmolality, while osmolality and viscosity were important determinants for tubular hydrostatic pressure and kidney exposure. The low diuretic effect of dimeric contrast media in combination with their high intrinsic viscosity resulted in a high viscosity within the tubular fluid. In comparison to monomeric contrast media, this led to a higher increase in tubular pressure, to a reduction in glomerular filtration rate and tubular flow and to an increase in kidney exposure. The presented kidney model can be implemented into whole body physiologically based pharmacokinetic models and extended in order to simulate the renal excretion of lipophilic drugs which may also undergo active secretion and reabsorption.

## Introduction

Hydrophilic agents like contrast media and osmotic diuretics are typically characterized by a predominant distribution in extracellular compartments and renal excretion by passive glomerular filtration (Better et al., 1997; Katzberg, 1997). Iodinated contrast media are commonly used in diagnostic radiology (Singh and Daftary, 2008). Currently used contrast media are based on a triiodinated benzene ring and can be classified as monomeric (i.e., containing one triiodinated benzene ring) and dimeric (containing two of these rings). Contrast media can be ionic and non-ionic substances, so that the ratio of iodine atoms per osmotically active particles (contrast medium molecules or ions) of commonly used contrast media ranges from 1.5 for ionic monomeric contrast media to 6 for non-ionic dimeric contrast media. The properties ionicity, osmolality, and viscosity are important characteristics that affect the safety of contrast media. Adverse effects became rare after non-ionic, low-osmolar contrast media came into use (Katzberg, 1997; Persson, 2006). The possible influence of osmolality and viscosity on the incidence of contrast-induced nephropathy, a rare but serious complication, has recently been discussed (Seeliger et al., 2012).

Currently employed contrast media are not reabsorbed from the tubules due to their hydrophilicity (low membrane permeability) and the absence of effective transporters.

The water reabsorption from the tubules is driven by an osmotic gradient between tubular and interstitial fluids. In the presence of contrast media, the osmolality of the tubular fluid is increased which leads to a decrease of water reabsorption (osmotic diuresis). The tubular concentration process is thus influenced by the osmolality of the contrast media. Given in equivalent iodine doses, monomeric contrast media cause a higher osmo-diuretic effect than dimeric contrast media and ionic contrast media cause a higher osmo-diuretic effect than non-ionic contrast media. Depending on the diuretic effect and the intrinsic viscosity of the contrast medium, the viscosity of the tubular fluid increases during urine concentration since fluid viscosity increases exponentially with the contrast medium concentration (Jost et al., 2010). This increased viscosity may subsequently lead to reduction of the glomerular filtration rate (Ueda et al., 1992; Seeliger et al., 2010), increase of the hydrostatic pressure within nephron tubules (Ueda et al., 1993) and, ultimately, retention of contrast media within the kidney (Jost et al., 2009, 2010).

In order to quantitatively investigate the relationship between substance properties of contrast media and their kidney exposure and renal excretion, a physiologically based kidney model was developed in the present study. The model should be able to consider the influence of osmolarity and viscosity of the contrast media on its concentration in the nephron tubules, the viscosity of tubular fluid, and the flow of tubular fluid.

## Materials and Methods

### Model structure

**Figure 1:**
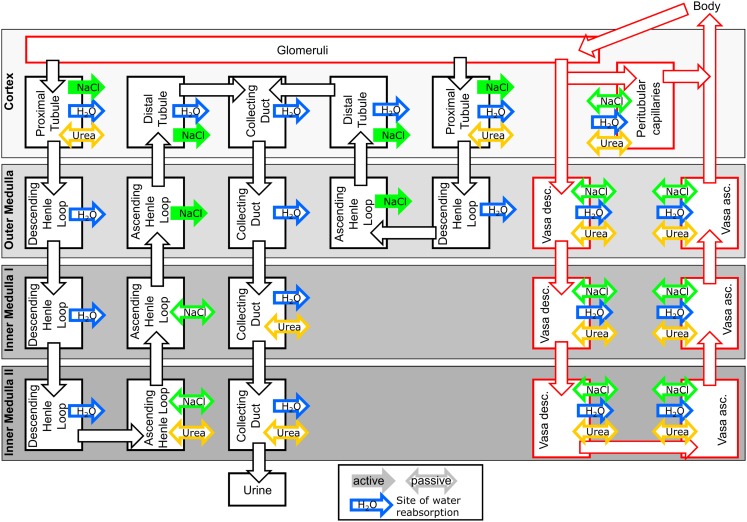
**Scheme of the kidney model**. The interstitial space compartments of the four kidney regions *Cortex* to *Inner Medulla II* are represented as shaded rectangles. Tubule lumen compartments are represented as black boxes and tubular fluid flow is represented by black arrows. Plasma compartments and plasma flow are represented by red boxes and red arrows, respectively. Sites of water reabsorption or uptake, sodium transport, and urea transport are indicated by blue, green, and yellow arrows, respectively. Solid arrows indicate active transport, open arrows indicate facilitated transport (passive diffusion) of NaCl or urea.

The kidney model structure is divided into four regions along the cortico-medullary axis: Cortex, Outer Medulla, Inner Medulla I, and Inner Medulla II (cf. Figure 1 for a scheme of the model structure). The inner medulla is divided into two regions to account for the osmolality gradient within the inner medulla (Layton et al., 2009). Each region is further subdivided into homogenous, well stirred tubular lumen, interstitial space, and blood plasma compartments (cf. Figure 1). The model consists of two types of tubular lumen compartments representing short loop nephrons (bending in Outer Medulla) and long loop nephrons (bending in Inner Medulla II), respectively.

A requirement of the model is to adequately describe the concentration and viscosity changes of hydrophilic compounds along the different tubule segments of the nephron. The largest fraction of fluid filtered in the glomeruli is reabsorbed from the tubular system (Landwehr et al., 1968; Sands and Layton, 2009). Hydrophilic compounds like contrast media or osmotic diuretics are not reabsorbed across the nephron epithelium due to their low membrane permeability and the absence of an effective transporter (Better et al., 1997; Katzberg, 1997). Their tubular concentration therefore depends on the degree of water reabsorption across the nephron epithelium. This water reabsorption is driven by the osmotic gradient across the nephron epithelium (Layton et al., 2009; Sands and Layton, 2009). Beside the administered hydrophilic agent, endogenous sodium and its accompanying anions as well as urea are considered within the model as the most important constituents of this gradient (Sands and Layton, 2009).

The concentration changes within the tubular lumen compartments are described within the present model by the following equation for each of the substances sodium, urea, and hydrophilic agent (drug) in the absence of transport processes:

(1) $$V_{i}\frac{dC_{i}^{\text{tub}}}{dt}=q_{\text{in,}i}C_{i-1}^{\text{tub}}-q_{\text{out},i}C_{i}^{\text{tub}}$$

where $C_{i}^{\text{tub}}$ is the tubular concentration of sodium, urea, or drug within the tubular segment *i* of Volume *V_i_*. The index *i* increases from proximal tubule to collecting duct. The tubular fluid flow out of the tubular segment *i* (*q*_out_,*_i_*) is smaller than the tubular fluid flow into the segment *i* (*q*_in,*i*_) because of the water reabsorption flow from tubular segment *i* (*q*_reabs,*i*_):

(2) $$q_{\text{out,}i}=q_{\text{in},i}-q_{\text{reabs},i}$$

The water reabsorption flow from the tubular segment *i* is driven by the osmotic gradient between the tubular fluid of segment *i* and its surrounding interstitial space of the kidney region *j* (ranging from Cortex to Inner Medulla II). The osmotic gradient is established by sodium, urea, and the exogenous hydrophilic agent (index “drug”):

$$\begin{array}{rcll} {q_{\text{reabs}}}_{,i} & =R_{i}\cdot \left[2\cdot C_{j,\text{Na}}^{\mathrm{int}}+C_{j,\text{urea}}^{\mathrm{int}}+n\cdot C_{j,\text{drug}}^{\mathrm{int}}\right. & & \\ & \;\left.-\left(2\cdot C_{i,\text{Na}}^{\text{tub}}+C_{i,\text{urea}}^{\text{tub}}+n\cdot C_{i,\text{drug}}^{\text{tub}}\right)\right] & & \text{(3)} \end{array}$$

where $C_{j,\text{Na}}^{\mathrm{int}},C_{j,\text{urea}}^{\mathrm{int}},{\text{and C}}_{j,\text{drug}}^{\mathrm{int}}$ are the interstitial sodium, urea, and drug concentrations within the kidney region *j*. The parameter *n* is the number of osmotically active species of the drug (e.g., 1 for non-ionic contrast media and 2 for ionic contrast media). To account for the anions accompanying sodium (mainly chloride), the sodium concentration is multiplied by a factor of 2. The parameter *R_i_* is a measure for the water conductivity for the tubular segment. The parameter *q*_reabs, *i*_ is restricted to values between 0 and *q*_in, *i*_.

The tubular fluid flow into a tubular segment equals the tubular fluid flow out of its preceding segment:

(4) $$q_{\text{in, }i+\text{1}}=q_{\text{out,}i}$$

If an active transport of sodium is present (cf. Figure 1) in the tubular segment *i*, a first-order transport term with rate constant *k*_active,*i*_ is added to Eq. 1. Similarly, a first-order diffusion term proportional to the tubular-interstitial concentration gradient is added to Eq. 1 for each passive diffusion which is present for sodium or urea in the tubular segment *i* and the kidney region *j* (cf. Figure 1):

$$\begin{array}{rcll} V_{i}\frac{dC_{i}^{\text{tub}}}{dt} & =q_{\text{in,}i}C_{i-\text{1}}^{\text{tub}}-q_{\text{out,}i}C_{i}^{\text{tub}}-k_{\text{active,}i}C_{i}^{\text{tub}} & & \\ & \;-k_{\text{passive,}i}\left(C_{i}^{\text{tub}}-C_{j}^{\text{int}}\right) & & \text{(5)} \end{array}$$

Generally, the interstitial sodium, urea, and drug concentrations within a kidney region change due to active transport processes, passive diffusion, and water reabsorption from tubular segments within that region. Additionally, diffusional exchange between the interstitial space and the plasma of peritubular capillaries (cortex) and of vasa recta (outer and inner medulla), respectively, has to be taken into account. Thus, for the interstitial concentration within the cortex $\left(C_{\text{cortex}}^{\mathrm{int}}\right)$ the following general equation is used for each substance sodium, urea, and drug:

$$\begin{array}{llll} V_{\text{cortex}}^{\mathrm{int}}\frac{dC_{\text{cortex}}^{\mathrm{int}}}{dt} & =\overset{na_{\text{cortex}}}{\underset{i=1}{\sum }}k_{\text{active,}i}C_{i}^{\text{tub}} & & \\ & \;+\overset{nb_{\text{cortex}}}{\underset{i\text{ = 1}}{\sum }}k_{\text{passive},i}\left(C_{i}^{\text{tub}}-C_{\text{cortex}}^{\mathrm{int}}\right) & & \\ & \;-\overset{n_{\text{cortex}}}{\underset{i=1}{\sum }}q_{\text{reabs,}i}\cdot C_{\text{cortex}}^{\mathrm{int}} & & \\ & \;-P^{\text{peritub}}S^{\text{peritub}}\left(C_{\text{cortex}}^{\mathrm{int}}-C^{\text{peritub}}\right) & & \text{(6)} \end{array}$$

The number of active transport processes and sites of passive diffusion within the cortex for the respective substance are designated as *na*_cortex_ and *nb*_cortex_, respectively and the number of water reabsorption sites as *n*_cortex_. The term *P*^peritub^*S*^peritub^ is the permeability-surface area product for the peritubular capillaries and *C*^peritub^ is the concentration of sodium, urea, or drug within the peritubular capillaries.

And analogously for the interstitial concentrations in the medullary region *j* (Outer Medulla to Inner Medulla II):

$$\begin{array}{rcll} V_{j}^{\mathrm{int}}\frac{dC_{j}^{\mathrm{int}}}{dt} & =\overset{na_{\text{j}}}{\underset{i=1}{\sum }}k_{\text{active,}\;i}\;C_{i}^{\text{tub}}+\overset{nb_{\text{j}}}{\underset{i=1}{\sum }}k_{\text{passive,}\;i}\left(C_{i}^{\text{tub}}-C_{j}^{\text{int}}\right) & & \\ & \;-\overset{n_{j}}{\underset{i=1}{\sum }}q_{\text{reabs,}\;i}\cdot C_{j}^{\mathrm{int}}-P_{k}^{\text{DVR}}S_{k}^{\text{DVR}}\left(C_{j}^{\mathrm{int}}-C_{k}^{\text{DVR}}\right) & & \\ & \;-P_{l}^{\text{AVR}}S_{l}^{\text{AVR}}\left(C_{j}^{\mathrm{int}}-C_{l}^{\text{AVR}}\right) & & \text{(7)} \end{array}$$

The number of active and passive transport processes for the respective substance present within the kidney region *j* are designated as *na_j_* and *nb_j_*, respectively and the number of water reabsorption sites as *n_j_*. The parameters $P_{k}^{\text{DVR}}S_{k}^{\text{DVR}}$ and $P_{l}^{\text{AVR}}S_{l}^{\text{AVR}}$ are permeability-surface area products for the descending vasa recta (DVR) or ascending vasa recta (AVR) capillaries of the vasa recta segment *k* and *l*, respectively. The concentration of sodium, urea, or drug in the DVR segment *k* and the AVR segment *l* is designated by $C_{k}^{\text{DVR}}\;\text{and}\;\;C_{l}^{\text{AVR}},$ respectively.

The concentrations within the peritubular plasma compartment are described by the following equation for each substance sodium, urea, and drug:

$$\begin{array}{llll} V^{\text{peritub}}\frac{dC^{\text{peritub}}}{dt} & =u_{\text{in,}\;\text{peritub}}\cdot C^{\text{glomeruli}}-u_{\text{out,}\;\text{peritub}}\cdot C^{\text{peritub}} & & \\ & \;+\overset{n_{\text{cortex}}}{\underset{i=1}{\sum }}q_{\text{reabs,}\;i}\cdot C_{\text{cortex}}^{\mathrm{int}} & & \\ & \;+P^{\text{peritub}}S^{\text{peritub}}\left(C_{\text{cortex}}^{\mathrm{int}}-C^{\text{peritub}}\right) & & \text{(8)} \end{array}$$

The parameter $u_{in,peritub}$ is the plasma flow into the peritubular compartment calculated from the renal plasma flow (RPF), the glomerular filtration rate for short and long loops (GFR_short_ and GFR_long_, respectively) and the fraction of peritubular plasma flow *f*_peritub_:

(9) $$u_{\text{in,}\;\text{peritub}}=f_{\text{peritub}}\left(\text{RPF}-\text{GF}{\text{R}}_{\text{short}}-\text{GF}{\text{R}}_{\text{long}}\right)$$

The peritubular plasma compartment takes up all fluid reabsorbed from cortical nephron segments:

(10) $$u_{\text{out,}\;\text{peritub}}=u_{\text{in,}\;\text{peritub}}+\overset{n_{\text{cortex}}}{\underset{i=1}{\sum }}q_{\text{reabs,}\;i}$$

The concentrations within DVR compartments are calculated by the following equation:

$$\begin{array}{rcll} V_{k}^{\text{DVR}}\frac{dC_{k}^{\text{DVR}}}{dt} & =u_{\text{in,}\;k}\cdot C_{k-\text{1}}^{\text{DVR}}-u_{\text{out,}\;k}\cdot C_{k}^{\text{DVR}} & & \\ & \;+P_{k}^{\text{DVR}}S_{k}^{\text{DVR}}\left(C_{j}^{\mathrm{int}}-C_{k}^{\text{DVR}}\right) & & \text{(11)} \end{array}$$

Since the plasma within the DVR enters high osmolal inner medulla, fluid is reabsorbed from the DVR which reduces the plasma flow rate out of the DVR segment *k* (*u*_out, k_):

(12) $$u_{\text{out,}\;k}=u_{\text{in,}\;k}-u_{\text{reabs,}\;k}$$

The fluid flow reabsorbed from the DVR (*u*_reabs, *k*_) is calculated in the same way as the water reabsorption from the tubules, cf. Eq. 3:

$$\begin{array}{rcll} u_{\text{reabs,}\;k} & =R_{k}\cdot \left[2\cdot C_{j\text{,}\;\text{Na}}^{\mathrm{int}}+C_{j\text{,}\;\text{urea}}^{\mathrm{int}}+n\cdot C_{j\text{,}\;\text{drug}}^{\mathrm{int}}\right. & & \\ & \;\left.-\left(2\cdot C_{k\text{,}\;\text{Na}}^{\text{DVR}}+C_{k\text{,}\;\text{urea}}^{\text{DVR}}+n\cdot C_{k\text{,}\;\text{drug}}^{\text{DVR}}\right)\right] & & \text{(13)} \end{array}$$

The concentrations in the AVR compartments are described by the following equation:

$$\begin{array}{rcll} V_{l}^{\text{AVR}}\frac{dC_{l}^{\text{AVR}}}{dt} & =u_{\text{in,}\;l}\cdot C_{l-\text{1}}^{\text{AVR}}-u_{\text{out,}\;l}\cdot C_{l}^{\text{AVR}}+\overset{n_{j}}{\underset{i=1}{\sum }}q_{\text{reabs,}\;i}\cdot C_{j}^{\text{int}} & & \\ & \;+P_{l}^{\text{AVR}}S_{l}^{\text{AVR}}\left(C_{j}^{\mathrm{int}}-C_{l}^{\text{AVR}}\right) & & \text{(14)} \end{array}$$

Since the number ratio AVR/DVR is approximately two (Pallone et al., 1994; MacPhee and Michel, 1995), $S_{l}^{\text{AVR}}=2\cdot S_{k}^{\text{DVR}}$was used in the present study.

The AVR segment *l* takes up the fluid reabsorbed from the corresponding DVR segment *k* as well as from all water permeable nephron segments within its kidney region:

(15) $$u_{\text{out,}\;l}=u_{\text{in,}\;l}+u_{\text{reabs,}\;k}+\overset{n_{\text{j}}}{\underset{i=1}{\sum }}q_{\text{reabs,}\;i}$$

The cellular space of the kidney is not structurally represented within the model, since cellular uptake via diffusion of hydrophilic drugs and contrast media is negligible (Better et al., 1997; Katzberg, 1997). Cellular uptake of contrast media can occur via vacuolization (Dobrota et al., 1995), but this process is not taken into account. In order to calculate tissue concentrations (mean concentrations weighted according to compartment volumes) of urea and sodium, cellular contributions are accounted for in the following way: Fixed values for cellular sodium concentrations are assumed: 25 mM in Inner Medulla II and 15 mM elsewhere (Beck et al., 1988). Cellular urea concentrations within a kidney region are assumed to equal the respective interstitial concentrations (Neuhofer and Beck, 2005). The cellular volumes are given in Table 1.

**Table 1:** **Compartment volumes used in the model as calculated from absolute volumes of kidney zones (Pfaller and Rittinger, 1980), the fractions of the respective kidney structures (Rasch and Dørup, 1997), the fraction of long loop nephrons (Jamison, 1987), and the hematocrit in renal circulation (Rasmussen, 1973)**.

		Volume (ml)
Cortex	Glomerular plasma	0.0772
	Peritubular capillaries	0.206
	Interstitial space	0.0664
	Proximal tubule short	0.172
	Proximal tubule long	0.0737
	Distal tubule short	0.0383
	Distal tubule long	0.0164
	Collecting duct	0.0548
	Cellular space	1.03
Outer medulla	Vasa descendens	0.0442
	Vasa ascendens	0.0442
	Interstitial space	0.0392
	Descending Henle’s loop short	0.0787
	Ascending Henle’s loop short	0.0392
	Descending Henle’s loop long	0.0337
	Ascending Henle’s loop long	0.0168
	Collecting duct	0.0488
	Cellular space	0.50
Inner medulla I	Vasa descendens	0.00318
	Vasa ascendens	0.00318
	Interstitial space	0.00467
	Descending Henle’s loop	0.00553
	Ascending Henle’s loop	0.00553
	Collecting duct	0.00213
	Cellular space	0.0139
Inner medulla II	Vasa descendens	0.00287
	Vasa ascendens	0.00287
	Interstitial space	0.0076
	Descending Henle’s loop	0.0038
	Ascending Henle’s loop	0.0038
	Collecting duct	0.0016
	Cellular space	0.0157

**Figure 2:**
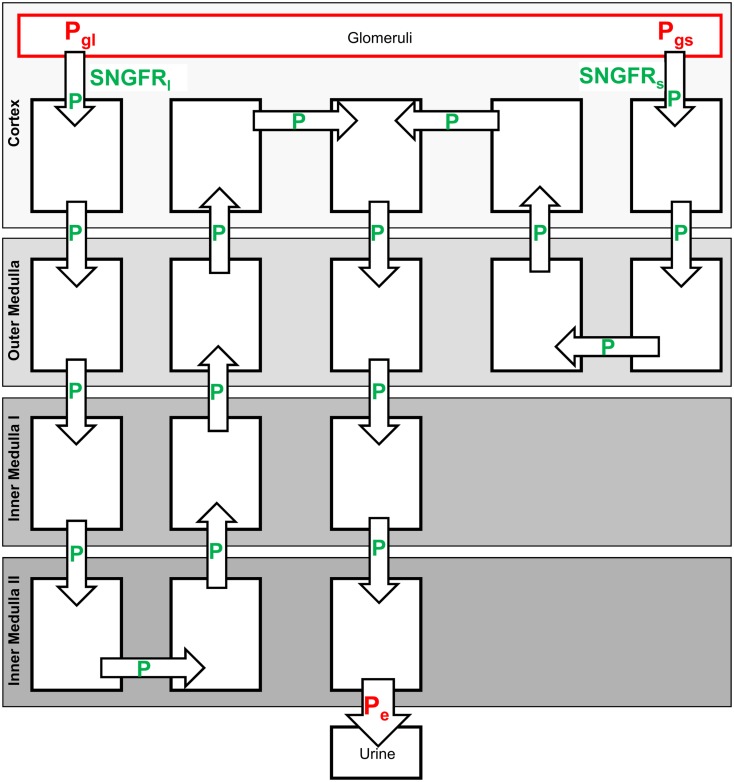
**Single nephron sub-model used to calculate hydrostatic pressures along the tubules and the SNGFR**. The parameters indicated in green are calculated from the Eqs 19–21, cf. text. The parameters indicated in red are set to fixed values.

As described above, administration of contrast media can lead to an increase in the viscosity of tubular fluid and, thus, affect urine flow and the rate of elimination. In order to account for such changes of tubular fluid flow, Hagen–Poiseuille type equations are used to calculate tubular pressure within a sub-model that represents a single short and long loop nephron (cf. Figure 2). A sub-model representing single nephrons is used in order to use the single nephron radius within the Hagen–Poiseuille type equations. Non-compliant tubes are assumed in the model. The viscosity of the tubular fluid is calculated in dependence of contrast agent concentration (cf. Model Evaluation and Simulation of Contrast Media). Fluid reabsorption flows and fluid flow along the single nephrons are calculated by dividing the respective flows from the total kidney model by the number of nephrons.

The collecting duct flow within the single nephron representation is increased in order to take into account joining of collecting ducts along the cortico-medullary axis. The numbers of collecting ducts per nephron used in the model are given in Table 2.

**Table 2:** **Used predefined physiological parameters**.

Total number of nephrons (both kidneys)	76000^a^
Fraction of long loop nephrons	30%^b^
Renal plasma flow (RPF; ml/min)	5.06^c^
Fraction peritubular plasma flow (*f*_peritub_)	85%^d^
Glomerular filtration rate at physiological steady state (GFR_0_; ml/min)	1.31^e^
Hematocrit in cortex and outer medulla	0.38^f^
Hematocrit in inner medulla	0.23^f^
Permeability of AVR for sodium, urea, and drug ($P_{l}^{\text{AVR}}$; cm/s)	120E−5^g^
Permeability of DVR for sodium, urea, and drug ($P_{k}^{\text{DVR}}$; cm/s)	76E−5^g^
Permeability of DVR for urea (P^DVR^ *k* 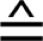 DVR in outer medulla; cm/s)	360E−5^g^
Permeability of peritubular capillaries for sodium, urea, and drug (*P*^peritub^; cm/s)	120E−5^h^
Surface area of peritubular capillaries (*S*^peritub^; cm^2^)	1.36E3^i^
Water conductivity of DVR [*R* (*k* 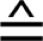 DVR in outer medulla); L^2^/(min μmol)]	2.2E−8^j^
Water conductivity of DVR [*R* (k 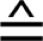 DVR in inner medulla I); L^2^/(min μmol)]	2.2E−8^j^
Water conductivity of DVR [*R* (*k* 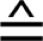 DVR in inner medulla II); L^2^/(min μmol)]	3.8E−9^j^
Systemic sodium plasma concentration (mmol/L)	150^k^
Systemic urea plasma concentration (mmol/L)	6^k^
Pressure at the end of collecting duct (*P*_e_; mmHg)	3^l^
Effective glomerular resistance (*R*_glom_; mmHg min/L)	2.205E9^m^
Pressure in glomerular capillaries, short loop nephrons (*P*_gs_; mmHg)	44.2^n^
Pressure in glomerular capillaries, long loop nephrons (*P*_gl_; mmHg)	47.2^o^
Number of collecting ducts per short loop nephron in cortex	1/4^p^
Number of collecting ducts per nephron in outer medulla	1/6^p^
Number of collecting ducts per nephron in inner medulla I	1/12^q^
Number of collecting ducts per nephron in inner medulla II	1/310^q^

*^a^Knepper et al., 1977; ^b^Jamison, 1987; ^c^calculated from systemic hematocrit 0.45 and renal blood flow 9.2 ml/min (Davies and Morris, 1993); ^d^Navar et al., 2008; ^e^Davies and Morris, 1993; ^f^Rasmussen, 1973; ^g^values for sodium and urea are taken from literature (Pallone et al., 1994), same values are used for the hydrophilic drug; ^h^same value used as for AVR; ^i^Crone and Levitt, 1984; ^j^estimated from hydraulic conductivity of DVR 1.6E−6 cm/s/mmHg (Pallone et al., 2003) and dimensions of DVR (Zimmerhackl et al., 1985); ^k^Atherton et al., 1970; ^l^Jensen et al., 1981; ^m^adjusted so that the start value for the single nephron glomerular filtration rate times number of nephron matches total glomerular filtration rate at physiological steady state (GFR_0_); ^n^Brenner et al., 1971; ^o^adjusted, so that the start value of the single nephron glomerular filtration rate for long loop nephrons equals that for short loop nephrons (Ericson et al., 1982; Sjöquist et al., 1984); ^p^Kainer, 1975; the number per long loop nephron (1/1.71) is calculated by the number of collecting ducts per short loop nephron in cortex (1/4) and the fraction of long loop nephrons (30%) and is matching the literature value of 1/2; ^q^estimated from Han et al. (1992)*.

The following Hagen–Poiseuille type equation is used, which takes into account fluid reabsorption that is assumed to be proportional to tubular flow rate (Macey, 1965; Jensen and Steven, 1979):

(16) $$P_{x}=P_{0}-\frac{8\cdot \eta \cdot \text{qs}{\text{n}}_{\text{in}}}{\pi \cdot r^{4}\cdot A}\left(1-e^{-A\cdot x}\right)$$

*P_x_* is the hydraulic pressure at position *x*, η is the viscosity of the tubular fluid, *r* is the tubular radius, qsn_in_ is the flow into the tubule of the single nephron representation and *A* is a parameter. Non-compliant tubules are assumed, i.e., the radius *r* is constant for each tubular segment during the simulation.

Since the fluid reabsorption for each tubular segment is known (calculated from the osmotic gradient) the parameter *A* can be calculated using

(17) $$\text{qs}{\text{n}}_{\text{out}}=\text{qs}{\text{n}}_{\text{in}}\cdot e^{-A\cdot L}$$

and

(18) $$\text{qs}{\text{n}}_{\text{out}}=\text{qs}{\text{n}}_{\text{in}}-\text{qs}{\text{n}}_{\text{reabs}}$$

where *L* is the length of a tubular segment, qsn_reabs_ is the fluid reabsorption flow from the tubular segment and qsn_out_ is the fluid flow at the end of the tubular segment.

Substituting *A* into Eq. 16 and setting *x* = *L*, gives the following equation which was used to calculate the pressures at the beginning (*P_m−1_*) and end (*P_m_*) of the tubular segments from which fluid reabsorption occurs:

(19) $$P_{m-1}=P_{m}+\frac{8\cdot \eta \cdot L\cdot \text{qs}{\text{n}}_{\text{reabs}}}{\pi \cdot r^{4}\cdot \ln\left(\frac{\text{qs}{\text{n}}_{\text{in}}}{\text{qs}{\text{n}}_{\text{in}}\text{ - qs}{\text{n}}_{\text{reabs}}}\right)}$$

For tubular segments without fluid reabsorption the Hagen–Poiseuille equation is used:

(20) $$P_{m-1}=P_{m}+\frac{8\cdot \eta \cdot L\cdot \text{qs}{\text{n}}_{\text{in}}}{\pi \cdot r^{4}}$$

The pressure at the end of the collecting duct *P*_e_ is assumed to be controlled by the pelvis/ureter and is set to a fixed value in the model. Upstream pressures are calculated using Eqs 19 and 20. Additionally, the pressures in the glomerular capillaries belonging to short and long loop nephrons *P*_gs_ and *P*_gl_, respectively, are assumed to be constant. Using these pressures, the single nephron glomerular filtration rates (SNGFR; fluid flow at the beginning of proximal tubules) of the short and long loop single nephron representations SNGFR*_s_* and SNGFR*_l_*, respectively, are calculated via the following equation:

(21) $$\text{SNGF}{\text{R}}_{s}=\frac{P_{\text{gs}}-P_{\text{ls}}}{R_{\text{glom}}}\;\;\text{and}\;\;\text{SNGF}{\text{R}}_{l}=\frac{P_{\text{g}l}-P_{\text{1}l}}{R_{\text{glom}}}$$

where *P*_1s_ and P_1l_ are the pressure at the beginning of the proximal tubule for the short and long loop nephrons, respectively, and *R*_glom_ is an effective hydraulic resistance of the glomeruli.

The total GFR of short and long loop nephrons (GFR_short_ and GFR_long_) of the kidney model shown in Figure 1 is given by the product of the number of short or long loop nephrons and the corresponding SNGFR_s/*l*_. The start value for SNGFR_s/*l*_ is calculated by the glomerular filtration rate of the physiological steady state (GFR_0_) and the number of nephrons (cf. Table 2).

Since the simulation software MoBi^®^ currently does not support differential algebraic equations, the algebraic Eqs 19–21 are implemented using stiff ODE equations adjusting the state variable to the algebraic equation. The algebraic condition

(22) $$y=F\left(x_{1},\;x_{2},\;\ldots x_{n}\right)\Leftrightarrow 0=y-F\left(x_{1},\;x_{2},\;\ldots x_{n}\right)$$

is thus implemented as

(23) $$\frac{dy}{dt}=\frac{\left(-y+F\left(x_{1},\;x_{2},\;\ldots x_{n}\right)\right)}{p}$$

with *p* = 0.1 min.

Within the current study, the rat body was represented by two compartments. One compartment represents the plasma volume taking up the administered dose of drugs or contrast media. From this compartment the substances are transported to the kidney by plasma flow. The second compartment represents the residual extracellular space.

### Parameterization of the physiological kidney model

In order to obtain *a priori* values for the numerous physiological parameters, a literature search was conducted. The volumes of the different model compartments are given in Table 1. The volumes are calculated from absolute volumes of kidney zones (Pfaller and Rittinger, 1980), the fractions of the respective kidney structures (Rasch and Dørup, 1997), the fraction of long loop nephrons and the hematocrit in renal circulation (cf. Table 2).

Further physiological kidney parameters used in the model are given in Table 2.

The dimensions of the nephron segments used in the single nephron sub-model which is used in order to calculate the tubular pressures are given in Table 3.

**Table 3:** **Radii and lengths of tubular segments used within the single nephron representation in order to calculate tubular pressures and SNGFR**.

	Radius *r* (μm)	Length *L* (mm)
Cortex: proximal tubule (short and long)	10.5^a^	6.2^e^
Outer medulla: descending Henle’s loop (short and long)	8.75^b^	2^f^
Inner medulla I: descending Henle’s loop	8^c^	2^f^
Inner medulla II: descending Henle loop	8^c^	2^f^
Inner medulla II: ascending Henle’s loop	8^c^	2^f^
Inner medulla I: ascending Henle’s loop	8^c^	2^f^
Outer medulla: ascending Henle’s loop (short and long)	9.5^c^	2^f^
Cortex: distal tubule (short and long)	9.5^a^	2^f^
Cortex: collecting duct	10^d^	1^f^
Outer medulla: collecting duct	10.5^d^	2^f^
Inner medulla I: collecting duct	12^d^	2.5^f^
Inner medulla II: collecting duct	14^d^	2.5^f^

*^a^Rasch and Dørup, 1997; ^b^the outer stripe of outer medulla contains the proximal straight tubule, thus a weighted mean of proximal tubule radius and descending Henle’s loop radius was used; ^c^Morgan and Berliner, 1968; Kainer, 1975; Kone et al., 1984; ^d^Knepper et al., 1977; ^e^Wahl and Schnermann, 1969; ^f^estimated from literature values of tubular segments lengths (Kainer, 1975) and kidney region dimensions (Kriz, 1967; Knepper et al., 1977)*.

The parameterization for the two compartmental representation of the rat body used within the current study was derived from the physiology database of the software PK-Sim (Willmann et al., 2003). The following parameters were used: 2.41 ml for the plasma volume taking up the administered dose of drug, 44.6 ml for the volume of the residual extracellular space and 0.02365 L/min for the total body plasma flow.

### Identification of missing parameters

Parameters that could not be obtained or estimated *a priori* from the literature were identified by fitting simultaneously to a set of experimental data for the physiological steady state, i.e., to properties without administration of drugs or contrast media. These data were: (a) cortico-medullary tissue concentration gradients of sodium and urea shown in Figure 4 (Atherton et al., 1970), (b) fluid reabsorption in tubular segments shown in Figure 5A (Landwehr et al., 1968), (c) urine flow for normal hydration state, 5.6 μL/min (Atherton et al., 1968), and (d) sodium as well as urea urine concentrations for a normal hydration state shown in Figure 5B (Atherton et al., 1968). The complete list of fitted parameters is given in Table 4 together with the respective parameter values.

**Table 4:** **Parameter values identified using experimental data on sodium and urea kidney concentrations, fluid reabsorption, urine flow, and sodium as well as urea urine concentration for a normal hydration state**.

Parameter	Identified value
**WATER CONDUCTIVITY [L^2^/(min μmol)]**	
*R* (*i* 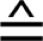 Proximal tubule)	1.2E−7
*R* (*i* 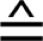 Descending Henle’s loop in outer Medulla)	2.7E−11
*R* (*i* 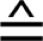 Descending Henle’s loop in inner Medulla I)	2.35E−10
*R* (*i* 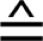 Descending Henle’s loop in inner Medulla II)	1.0E−12
*R* (*i* 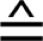 Distal tubule)	6.77E−8
*R* (*i* 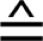 Collecting duct in cortex)	7.406E−9
*R* (*i* 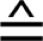 Collecting duct in outer medulla)	5.078E−9
*R* (*i* 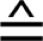 Collecting duct in inner medulla I)	1.164E−9
*R* (*i* 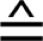 Collecting duct in inner medulla II)	4.443E−11
**RATE CONSTANTS SODIUM TRANSPORT (L/min)**	
k_active_ (*i* 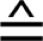 Proximal tubule)	2.48E−4
k_active_ (*i* 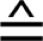 Ascending Henle’s loop in outer medulla)	7.87E−4
k_active_ (*i* 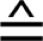 Distal tubule)	5.11E−4
**RATE CONSTANTS SODIUM DIFFUSION (L/min)**	
k_passive_ (*i* 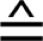 Ascending Henle’s loop in inner medulla II)	2.5E−3
k_passive_ (*i* 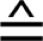 Ascending Henle’s loop in inner medulla I)	2.5E−3
**RATE CONSTANTS UREA DIFFUSION (L/min)**	
k_passive_ (*i* 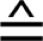 Proximal tubule)	8.2E−9
k_passive_ (*i* 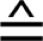 Ascending Henle’s loop in inner medulla II)	4.89E−6
k_passive_ (*i* 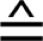 Collecting duct in inner medulla I)	1.39E−6
k_passive_ (*i* 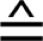 Collecting duct in inner medulla II)	4.45E−5
**SURFACE AREA DVR (cm^2^)**	
S (*j* 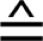 Outer medulla)	1.0E−3
S (*j* 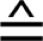 Inner medulla I)	1.0E−4
S (*j* 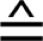 Inner medulla II)	1.0E−4

*The values for water conductivities and rate constants refer to the long loop nephrons. The values for short loop nephrons are obtained by scaling the values for the long loop nephrons by the ratio of the number of short loop nephrons to the number of long loop nephrons*.

### Model evaluation and simulation of contrast media

**Figure 3:**
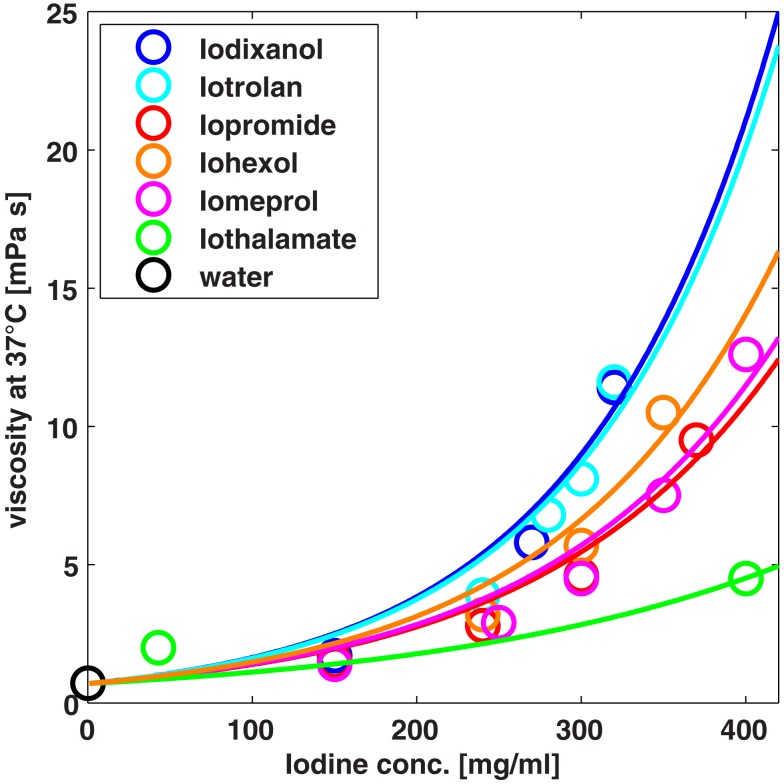
**Viscosity of different contrast media at different concentrations**. The exponential functions (solid lines) used to calculate the viscosity of tubular fluid are compared to experimental data (symbols).

The model established using physiological steady state data is evaluated by predicting effects of osmotic mannitol diuresis and administration of contrast media. In other words, model simulations are compared to experimental data after administration of mannitol or contrast media without any adjustment of model parameters. The viscosities in tubular segments used in the model Eqs 19 and 20 are calculated from an empirical exponential function for each contrast medium (cf. Figure 3). The functions are obtained by fitting the exponent to experimental fluid viscosity vs. contrast media concentration data taken from the product brochures of the respective vendors.

For mannitol simulations it was assumed that mannitol does not alter the viscosity, i.e., the viscosity of water was used for the tubular fluid.

The effect of mannitol administration on the sodium and urea concentration gradients and urine flow was predicted and compared to experimental data from the literature (Atherton et al., 1968), where a priming injection of 0.15 g mannitol (15 g/100 ml solution) followed by an infusion of 6.75 g mannitol over 450 min was administered.

The model was further evaluated by predicting contrast media effects on urine flow and urine viscosity for the monomer iopromide and the dimer iodixanol (Seeliger et al., 2010). A urine density of 1 g/ml was used to convert the dynamic viscosities from model output to the experimental kinematic viscosities. Additionally, contrast media effects on proximal and distal tubular pressure (Ueda et al., 1993) and SNGFR (Ueda et al., 1992) are predicted for the monomer iohexol and the dimer iotrolan.

### Software

The model was implemented using the software MoBi^®^, Version 2.3, Bayer Technology Services, Leverkusen, Germany (Eissing et al., 2011). All optimizations and batch mode simulations for MoBi^®^ models were done using MATLAB^®^ (R2010b, The MathWorks Inc., Natick, MA, USA) and the MoBi Toolbox for MATLAB^®^ (Version 2.2, Bayer Technology Services, Leverkusen, Germany) or directly with MATLAB^®^ executable files exported from MoBi^®^.

## Results

### Model development – physiological steady state

**Figure 4:**
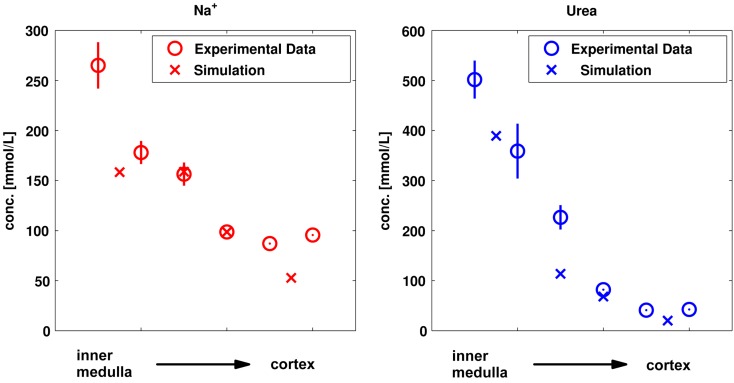
**Comparison of simulated and experimental sodium and urea kidney tissue concentrations (Atherton et al., 1970) used for parameter identification**. The simulated values for the four kidney regions are compared to the experimental values from six kidney sections (papillary tip, papillary base, inner medulla, outer medulla, inner cortex, and outer cortex).

The simulation results for the sodium and urea kidney concentrations are compared to the experimental tissue concentrations used for parameter identification in Figure 4.

The sodium and urea gradients for “normally” hydrated rats (which had free access to water and a urine osmolality of 800–1600 μosmol/g water; Atherton et al., 1970) are qualitatively described by the model. The sodium concentrations tend to be underestimated by the model in the cortex and in the innermost part of the inner medulla. Also the simulated urea concentration for the Inner Medulla I is lower than the reported experimental data.

**Figure 5:**
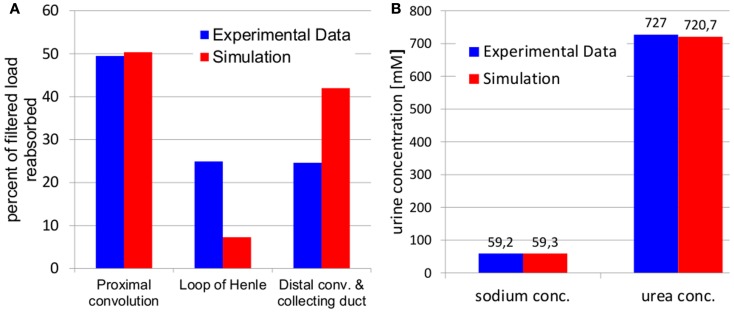
**Comparison of simulated fluid reabsorption (A) and sodium and urea urine concentrations (B) to experimental data used for parameter identification**. Experimental fluid reabsorption (Landwehr et al., 1968) and experimental urine concentrations (Atherton et al., 1968) are taken from literature.

The simulation results after parameter identification for tubular fluid reabsorption and for sodium as well as urea urine concentrations are given in Figure 5.

It can be seen, that the simulated fluid reabsorption is shifted from Henle’s loop to the distal tubule and collecting duct. However, the total fluid reabsorption is in excellent agreement with experimental data for the “normal” hydration state (5.6 μL/min urine flow in simulation as well as experiment; Atherton et al., 1968). Also the simulated sodium and urea urine concentration excellently match the experimental values for the “normal” hydration state. The corresponding identified parameter values are given in Table 4.

The rate constant for urea diffusion in the proximal tubule as well as the surface areas of the vasa recta were found to be very small (insensitive to further decrease for physiological steady state properties) by the parameter identification procedure.

### Model evaluation: Osmotic mannitol diuresis

**Figure 6:**
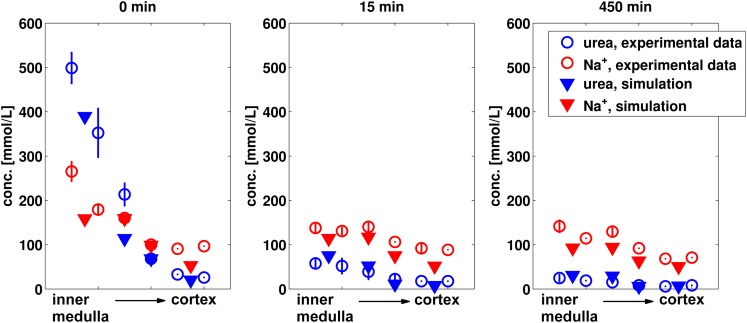
**Predicted sodium and urea kidney concentrations after 15 and 450 min of mannitol infusion in comparison to experimental data (Atherton et al., 1968)**. The values for non-diuretic control (0 min) are given for comparison. The simulated values for the four kidney regions are compared to the experimental values from six kidney sections (papillary tip, papillary base, inner medulla, outer medulla, inner cortex, and outer cortex).

**Figure 7:**
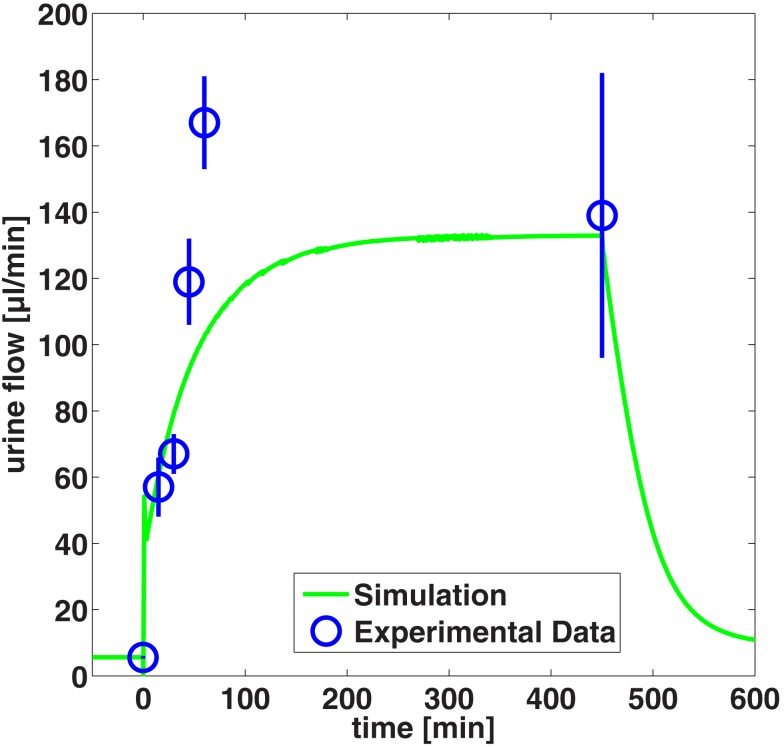
**Predicted urine flow during mannitol diuresis compared to experimental data (Atherton et al., 1968)**.

The established kidney model was evaluated by predicting the effect of administration of a priming injection of 0.15 g mannitol followed by an infusion of 6.75 g mannitol over 450 min. The predicted effect on sodium and urea concentration gradients as well as on urine flow is compared to experimental data from the literature (Atherton et al., 1968) in Figures 6 and 7 respectively.

As can be seen in Figure 6, the decrease of urea and sodium concentrations in the medulla are very well predicted. In particular the almost complete breakdown of the urea gradient during mannitol diuresis is quantitatively described by the model.

The overall level of urine flow increase during manitol diuresis is predicted very well, whereas the maximum urine flow after 45 and 60 min infusion is underestimated by the model (Figure 7).

### Model evaluation: Contrast media effects

**Figure 8:**
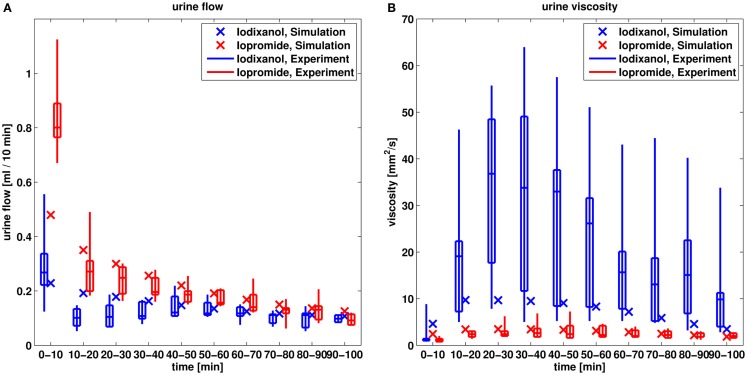
**Comparison of predicted urine flow (A) and urine viscosity (B) after administration of iodixanol and iopromide to experimental data (Seeliger et al., 2010)**. The box-whisker plots of the experimental data give the 5th, 25th, 50th, 75th, and 95th percentile.

In the next step, the model is evaluated by predicting contrast media effects taking into account changes of viscosity of the tubular fluid. The predicted effects on urine flow and urine viscosity after administration of the dimeric iodixanol and the monomeric iopromide are compared to experimental data in Figure 8.

The larger urine flow for the monomeric iopromide compared to the dimeric, lower osmolal iodixanol is well predicted by the model, although the simulated urine flow is underestimated for the initial 10 min for iopromide.

Also the larger increase of the urine viscosity of iodixanol compared to iopromide is predicted by the model. The maximum urine viscosity of iodixanol is underestimated by the model by a factor of approximately four. That means that the maximum urine concentration of iodixanol is underestimated by only a factor of 1.5, given the exponential viscosity vs. concentration relationship from Figure 3.

**Figure 9:**
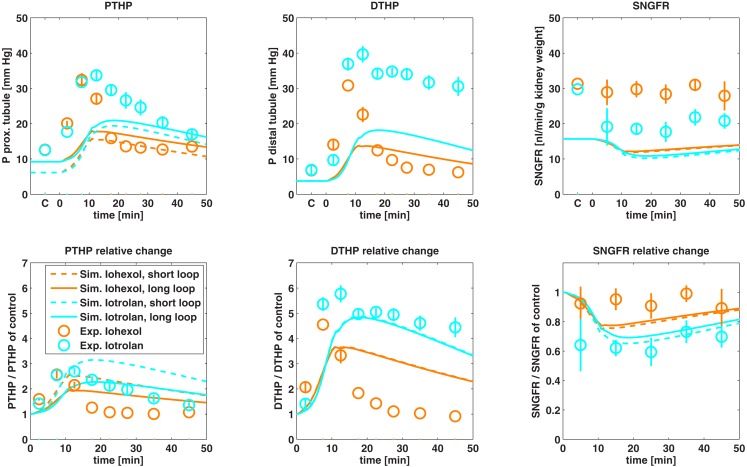
**Comparison of predicted proximal tubular hydrostatic pressure (PTHP), distal tubular hydrostatic pressure (DTHP) as well as single nephron glomerular filtration rate (SNGFR) after administration of iohexol and iotrolan to experimental data**. Control experiments are designated by *C* on the time axis. Experimental hydrostatic pressures are taken from Ueda et al. (1993) and experimental data on SNGFR are taken from Ueda et al. (1992). Absolute changes (first row) as well as changes relative to control (second row) are shown.

The predicted effects on proximal and distal tubular hydrostatic pressure as well as on SNGFR after administration of the monomer iohexol and the dimer iotrolan are compared to experimental data in Figure 9.

The model predicted an increase in proximal and distal tubular pressure during administration of the contrast agents that is qualitatively in agreement with the observed data and the differences between iotrolan and iohexol were qualitatively predicted. The absolute tubular pressures are underestimated by the model after contrast medium administration as well as for the control without contrast medium applied. After maximum tubular pressure is reached, the simulated pressure for iohexol decreases slower than experimentally observed. Otherwise, the relative pressure changes are in good agreement with experimental data. The simulated SNGFR is lower than the experimentally observed SNGFR by a factor of approximately two. The relative SNGFR change of the dimeric iotrolan is in good agreement with the experimental data, while the relative change for iohexol is overestimated by the model. Overall, the essential trends are described with the model.

### Comparison of typical iodinated contrast media

**Figure 10:**
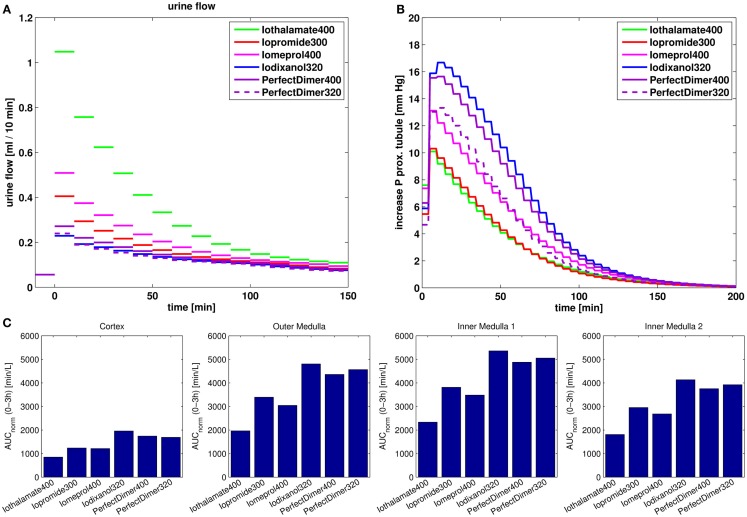
**Comparison of effects of different contrast media on urine flow (A), increase of hydrostatic pressure in the short loop proximal tubule (B) and kidney exposure measured as dose normalized area under the tissue concentration-time curve for 0–3 h post dosing (C)**.

With the validated model, the effects of osmolarity and viscosity of different contrast media on urine flow, hydrostatic tubular pressure, and kidney exposure are compared. As typical examples iopromide 300 (300 mg iodine/ml) and iomeprol 400 (400 mg iodine/ml), both non-ionic monomeric contrast media, iodixanol 320 (320 mg iodine/ml), a non-ionic dimeric contrast medium, and iothalamate 400 (400 mg iodine/ml), a ionic monomeric contrast medium were used. For comparison, also a hypothetical non-ionic “Perfect Dimer” with six iodine atoms per molecule and the same viscosity vs. iodine concentration relationsship as for the monomer iopromide was used. The simulation results for the different contrast media for a dose of 1.5 ml are compared to each other in Figure 10.

**Figure 11:**
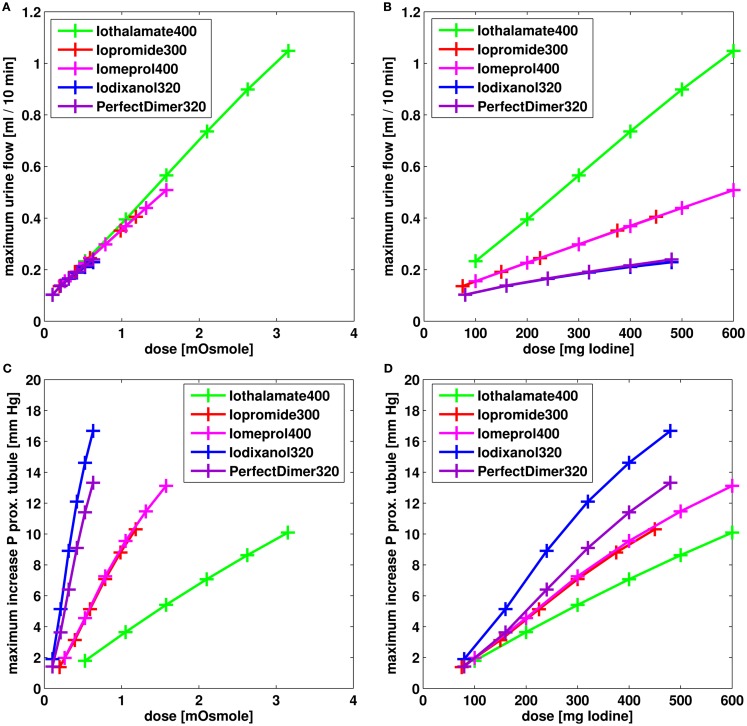
**Effect on urine flow (A,B) and increase of hydrostatic pressure in short loop proximal tubule (C,D) for different dosages of contrast media**. The dose is given in mOsmole of osmotic active particles **(A,C)** and in mg iodine **(B,C)**.

The effect of different dosages on the maximum urine flow and the maximum increase in hydrostatic pressure in proximal tubule is compared for the different contrast media in Figure 11.

The urine flow predominately depends on the dose measured in osmole (Figure 11A). Accordingly, the behavior of the “Perfect Dimer” is similar to iodixanol regarding urine flow (Figures 10A and 11A,B) and the three groups dimeric CM (“perfect dimer” and iodixanol), monomeric CM (iopromide and iomeprol), and monomeric ionic CM (iothalamate) are clearly separated if equivalent iodine dosages are considered (Figure 11B).

The increase in hydrostatic pressure depends on both, viscosity and dose in osmole. The exposure of “Perfect dimer” is more similar to iodixanol than to monomeric CM. The absolute difference is small.

It can be seen, that for exposure and especially for the increase in hydrostatic pressure, the reduction of viscosity for the perfect dimer does not lead to behavior/properties of a typical monomer.

## Discussion

A computational kidney model was developed in order to analyze the renal excretion and kidney exposure of hydrophilic substances, in particular iodinated contrast media. Regarding safety, the osmolality and viscosity are considered to be important properties of contrast media (Seeliger et al., 2012). The model was thus designed to describe the concentration and accompanying viscosity change of contrast media along the nephron tubules. The contribution of contrast media to tubular osmolality decreases fluid reabsorption and gives thus rise to a diuretic effect, which in turn influences tubular contrast medium concentration. Thus, a requirement of the model was to mechanistically describe the diuretic effect as well as changes in tubular flow due to viscosity changes in tubular fluid.

The model was developed using experimental data relevant for urine concentration. These data, for a normal hydration state, were: (a) the cortico-medullary sodium and urea concentration gradients, (b) water reabsorption data, (c) urine flow, and (d) sodium as well as urea urine concentration. The sodium and urea gradients could be qualitatively described by the model. Quantitatively, urea and in particular sodium concentrations were underestimated in the inner medulla. A possible reason for this mismatch might be the limited discretization along the cortico-medullary axis into only four kidney regions and the neglect of the length distribution of Henle’s loop within the inner medulla. Highly detailed models with a continuous representation of the cortico-medullary axis and with loop turns distributed along the cortico-medullary axis have been developed by Layton et al. (2004) which are able to predict the sodium and urea urine concentrations of moderately antidiuretic rats. These models were further extended to represent the radial organization of renal tubules and vessels (Layton, 2011; Layton et al., 2012). Other modeling studies testing several mechanisms of urine concentration have been published (Thomas et al., 2006; Edwards, 2010), however the urine concentrating mechanism is still not completely understood (Dantzler et al., 2011).

Cortical sodium concentrations are also underestimated by the model. The simulated sodium concentrations in the proximal tubules and in the cortical plasma or interstitial space are close to the systemic plasma concentration of 150 mM. Since the distal tubular and cortical collecting duct lumen together account for only less than 7% of the cortical volume, a possible explanation for the underestimation of the cortical tissue concentration is the representation of the cellular space: Either the fraction of cellular space is too large or the fixed sodium concentration in cellular space is too small in the present model, although the respective values were taken from literature. Detailed computational models describing solute and fluid transport for single tubule segments taking explicitly into account epithelial cells are described in literature (Weinstein, 2003; Weinstein et al., 2007; Weinstein and Krahn, 2010).

The validation by predicting the effects of mannitol and contrast media administration showed that the essential processes of water reabsorption and tubular flow in dependence of drug or contrast media concentrations are covered by the model. Due to the exponential concentration vs. viscosity relationship, small errors in concentrations lead to larger errors in the viscosity of the tubular fluid and urine. Thus, the predicted urine viscosity after administration of iodixanol was underestimated by a factor of up to four (cf. Figure 8B), while the simulated iodixanol concentration was underestimated only by a factor of approximately 1.5. Only the initial simulated iodixanol viscosity was larger than the observed viscosity possibly due to a missing time lag caused by the ureter which is not represented in model.

The simulated SNGFR is lower than the experimental SNGFR by a factor of approximately two after administration of contrast media as well as for the control. The reason for this is that the measured SNGFR does not match the literature values for the total GFR in the physiological steady state and for the number of nephrons used in the present model.

In the model, the effects of viscosity changes of the tubular fluid are described by transient changes in the glomerular filtration rate via pressure changes in non-compliant tubules. The assumption of non-compliant tubules with constant radii during diuresis is a limitation of the model. However, during diuresis, not only the tubular pressure increases but also the interstitial pressure due to the restricted distensibility of the kidney by the rather inelastic renal capsule (Garcia-Estan and Roman, 1989; Khraibi and Knox, 1989). Hence, the increase of tubular diameter during osmotic diuresis is smaller than for furosemide diuresis due to a larger increase in interstitial pressure (Cortell et al., 1973). A further model limitation is that any physiological mechanisms regulating the glomerular filtration rate and the renal blood flow, for example the tubuloglomerular feedback (Just, 2007), are not represented in the model. Mathematical models especially focusing on these mechanisms are available in literature (Holstein-Rathlou and Marsh, 1990; Feldberg et al., 1995; Thomas et al., 2006). Recently, a mathematical model taking into account tubuloglomerular feedback was used in order to analyze the mechanism of pressure-diuresis and pressure-natriuresis (Beard and Mescam, 2012). Another regulatory mechanism which is currently not represented in the model is the renin-angiotensin-aldosterone system, which influences GFR as well as sodium and water reabsorption (Kobori et al., 2007).

The present model was used to analyze and compare the effects of different classes of contrast media for the normal hydration state. The concentrations of a contrast medium within the tubules depend on its osmolality. The higher the osmolality, the higher is the diuretic effect and the lower is the contrast medium concentration in the tubular fluid. The low diuretic effect of dimeric contrast media in combination with their high intrinsic viscosity results in a high viscosity within the tubular fluid. In comparison to monomeric contrast media, this leads to a higher increase in tubular pressure, to a reduction in glomerular filtration rate and tubular flow and to an increase in kidney exposure.

The model allows the simulation of contrast media with hypothetic properties. In the present study a “Perfect Dimer” with six iodine atoms per molecule and a very low viscosity (same iodine concentration vs. viscosity relationship as iopromide) was compared to typical iodinated contrast media. It was found, that for the kidney exposure and especially for the increase in hydrostatic pressure, the reduction of viscosity for the perfect dimer does not lead to a behavior of a typical monomer. The reason for this is that the reduced osmolality causes a reduced diuretic effect which in turn increases tubular concentration and viscosity of the perfect dimer. The model focus was on the description of tubular concentrations, diuretic effect and tubular flow without taking regulation mechanism into account. Thus, effects of contrast media on, e.g., tubuloglomerular feedback and medullary blood flow, which also have been observed (Seeliger et al., 2007, 2012), cannot be described by the model.

Due to its physiological foundation, the kidney model for rats can be scaled to other species, especially to humans. In order to describe the renal excretion of general, lipophilic drugs, the model can be extended by an explicit representation of nephron epithelium and other cellular space. Since the drug concentration in tubular lumen, nephron epithelium, and interstitial space would be represented within such an extended model, it potentially could be used to estimate passive reabsorption of lipophilic drugs. Also active secretion and reabsorption process could be represented in such a model, which could then be used to describe the renal clearance of drugs within whole body physiologically based pharmacokinetic models.

## Conflict of Interest Statement

The authors are employed by Bayer, a company selling contrast media, in particular the iodinated contrast medium Ultravist^®^ (iopromide)
